# Treatment of Oral Infection in Its Relation to Systemic Disease

**Published:** 1914-08-15

**Authors:** Joseph Head

**Affiliations:** Philadelphia


### TREATMENT OF ORAL INFECTION IN ITS RELATION TO SYSTEMIC DISEASE.

BY JOSEPH HEAD, M.D., D.D.S., OF PHILADELPHIA.

The disease of pyorrhea alveolaris, in spite of its name, is not always associated with a flow of pus from the infected tooth sockets. As a matter of fact, pus does not appear in 25 percent. of the cases. The main symptoms are inflammation of the peridental membrane, bleeding of the gums at the slightest touch and tendency for the teeth to elongate and be sensitive to mastication. Finally, the gums separate from the roots of the teeth, forming pockets where salivary calculus and infection cause such centres of irritation and disintegration that the destruction of the teeth involved is only a question of time, unless the tartar is removed from the roots, and the mass of infection is destroyed, and the gum so stimulated to reattach itself to the root.

Pyorrhea alveolaris unquestionably has a systemic as well as a local origin. It is frequently associated with no particular systemic disturbance that either the physician or patient can distinctly note, and yet these apparently non-toxic cases, under skillful treatment, will almost invariably show systemic improvement of a subnormal condition, that up to the time of pyorrhea treatment had been unsuspected both by patient and physician. It is a common occurrence for a patient under treatment for pyorrhea to say, "I have not felt so well in years, and I can only attribute the improvement to the pyorrhea treatment." When this statement is made by patient after patient independently, and is associated with a gain in weight or rise in haemoglobin, from 67 to 93, or a disappearance of chronic constipation, etc., it indicates strongly that there are no real cases of non-systemic toxic pyorrhea. It strongly indicates also, that all foci of infection in the mouth, although some may be apparently well borne, are a source of toxemia.

A single blind fistula from an infected root may cause the most profound nervous depression which, being chronic, will only be recognized by its disappearance with the cure of the fistula. Osler, Goadby, Daland, Hunter, and Billings, I believe, have mentioned the bodily ailments that time will show to be directly traced to mouth infections. That mouth infection is, in a great majority of cases, associated with Bright's disease, diabetes, cardiac, hepatic, renal, and stomach disorders, has been known for a long time, but within recent years it has been shown that these disorders are, in many instances, ameliorated, and, at times, entirely eradicated by the removal of the infecting foci from the mouth. My experience in the treatment of pyorrhea alveolaris with its associated tooth infections, accords with these findings.

In the light of our present knowledge any dentist that puts in a bridge or crown that cannot be cleansed as thoroughly as any one of the natural teeth, or knowingly allows foci of infection to continue in the gums or root canals, is endangering his patients' health and life, and I have no doubt that such procedure in the near future will be accounted malpractice. Yet, to be sure that there are no infecting foci requires a discrimination that taxes the ability of the trained, conscientious dentist to the utmost. One canal of a molar pulp may have died, spreading infection throughout the system, while the rest of the pulp being alive may cause the tooth to appear normal.

Pulps under fillings, although alive, may have become infected from the decay prior to its removal and so cause a fistula, open or concealed, that on account of the living infected pulp is difficult to diagnosticate. However, there is no excuse for allowing obvious fistulas to continue to discharge their contents into the mouth or blood current.

When we come to pyorrhea that the great majority of dentists class as incurable, we have come to the great divide, for insomuch as dentists shall control and cure this great scourge, pyorrhea alveolaris, so shall they take their places in the dentistry of the future, and insomuch as they neglect this great source of human disease, they shall be relegated to the spheres of those who have unwisely attempted that which they are unfit to accomplish. For the cure of pyorrhea alveolaris has been more or less successfully accomplished for the last fifteen or twenty years.

While, as previously stated, pyorrhea is associated with various disturbances of which it may be the primal and continuing cause, such as gout, Bright's disease, diabetes, cardiac, hepatic, and renal disorders, such specific diseases as syphilis, gonorrhea, and tuberculosis by their debilitating effect on the tissues of the mouth, may so lower the vitality of the gums that infections that otherwise would be repudiated penetrate the peridental membrane, and by a subtle toxemia still further depress the general vital forces. This infection having penetrated the gums, forms a small abscess that partly strips away the peridental membrane from the tooth neck forming a pocket, which, once being formed, continues the disease even after the general systemic disorder may have been conquered. It is probable that some one of the numerous passing diseases, such as typhoid, measles, grip or even a bad cold, has temporarily lowered the physical resistance of the gum tissue to the point of permitting the ever present infection to penetrate and form the self-perpetuating pockets, thus leaving the pyorrhea to continue without apparent systemic cause. Where the systemic cause has passed and the pyorrhea has not created a new one, the disease may, perhaps, be successfully treated by local means and a permanent cure hoped for. But where pyorrhea is associated with serious general disorder, local treatment, important as it is, can only be held as a palliative, and must be supplemented by judicious systemic treatment.

The most important systemic treatment in the cure of pyorrhea consists in the discriminating use of vaccines that will produce antibodies in the blood as supplementary adjuncts to the proper local treatment. And yet important and valuable as the vaccine treatment unquestionably is, without the proper local treatment that removes infecting foci and stimulates the gums to heal and reattach themselves to the roots, vaccine treatment can only reduce the severity of the symptoms. It cannot effect a cure.

Up to within the last four or five years instrumentation that removed bacterial masses and tartar scales from the roots beneath the gums was the chief reliance for the cure of pyorrhea alveolaris. It is true that various medicaments, such as 75 percent. lactic acid or 50 percent. sulphuric acid were used with the idea of softening the scale and rendering it more easy of removal. The objection to these medicines is that when diluted by the fluids of the mouth they attack the enamel of the teeth more rapidly than the tartar. A number of dentists have been able to tighten single-rooted teeth partly by scaling and partly by splinting to adjacent firm teeth, but in cleansing the calcareous deposits from the bifurcation of roots only a few dentists have succeeded, and some of these by the ruthlessness of their instrumentation have at times caused unjustifiable injury to the cementum and pulpal vessels. It has also been noted that deep pockets occasionally appear around a tooth that is sensitive and loose during mastication, and yet the most careful manipulation of the scaler has failed to show the presence of the tartar scale. This seems to prove that the mere absence of tartar is not a sufficient circumstance to effect a re-attachment of the gums to the root. What we need is an efficient antiseptic solvent that will soften the tartar attachment to the root to expedite instrumentation, that will dissolve any microscopic portions inadvertently overlooked, an antiseptic that in destroying the bacteria within the adjacent gum tissue, will not depress but stimulate the tissue cells, so that they will form re-attachment of the gum to the root, thus causing the disappearance of the pocket, which is the self-perpetuating focus of infection. This medicine we have in the 20 percent. aqueous solution of bifluoride of ammonium with 10 percent. free hydrofluoric acid.

Some seven years ago it was my good fortune to discover that commercial hydrofluoric acid would disintegrate tartar with no microscopic action on either cementum or enamel of an extracted tooth. The poisonous action of this acid alone precluded its use as a therapeutic agent to be applied to the gums. Through an extensive series of experimentation it was proven that a 20 percent. solution of bifluoride of ammonium with 10 percent. hydrofluoric acid will disintegrate the tartar on a tooth and leave the tooth apparently unsoftened. This mixture has germicidal strength of 5.82 times pure carbolic acid, and on most mucous membrane is not escharotic.

I also use a 20 percent. solution of bifluoride of ammonium with only 5 percent. free hydrofluoric acid. This is valuable where there is excessive gum inflammation, as it can be applied without the pain which the first one or two applications of the ordinary solution is apt to occasion. When the inflammation is reduced the stronger solution can be painlessly and advantageously used. The solution of ammonium bifluoride may be injected once or twice a week into pockets around loose teeth, but care must be taken not to inject it into fresh cuts or the tissue, as such a procedure will cause great pain.

The patient should spit without rinsing the mouth with water, the saliva acting as a protection against irritation. After one or two injections the soreness and inflammation will largely disappear, and even the general symptoms of toxemia will be sometimes found to have abated. The tartar scale that could not be easily and painlessly removed at the first two sittings, will now tend to be so loosened that its thorough removal by scalers will be easy for both patient and dentist. After four or five applications, one week apart, black scales that have escaped the instruments will sometimes be found floating loose in the pockets, so that they can readily be picked out, and finally the root will become as smooth as velvet to the touc h of an instrument. When this stage has been reached it will be found that the scalers cannot be as deeply carried into the pyorrhea pockets as was easily possible in the beginning. To do so causes pain and a free flow of blood. This indicates that new granulations are forming, and these should not be ruthlessly broken up either by instrumentation or by injection of the bifluoride into them.

The solution should be gently flowed into the pockets each week, a procedure which results in continual healing and is not productive of pain. Careful exploration for tartar on the root within the pocket is always advisable. Re-attachment of the gum to the root, however, can be accepted as assurance that that portion of the root has been thoroughly cleansed, and so the scaling in that place should be discontinued, while the weekly applications of the bifluoride solution should alone be used. If, however, at the end of two or three months any of the pockets have not entirely healed, they should be re-explored with scalers and the treatment repeated as for a new case, although by this time most of the original pockets will have disappeared, and where they existed, the gums, with a slight contraction at the neck, will be found firmly adherent to the tooth. Teeth that have lost more than half of their gum attachment, under this treatment have become firm and comfortable to the action of normal mastication.

Loose teeth may be splinted together so as to give immobility, but this should always be done in such a manner as not to prevent thorough cleansing of the gum around their necks. Malocclusion should be remedied so that loose teeth may be relieved of the excessive pressure of mastication due to thickening of the inflamed peridental membrane. When a pocket has approached an apical foramen near enough to infect the pulp, the pulp should be destroyed and the root canals thoroughly sterilized and filled. This should be especially looked to in the case of molars where the pocket extends through the bifurcation and down to the tip of one of the roots.

It is sometimes excellent practice to cut the crown midway through the grinding surface through the juncture of the roots, finally extracting the diseased root with its side of the crown. This exposes the other root or roots so that they can be filled and cleaned as easily as a bicuspid. With upper molars, where the palatal root is necrosed, this operation is most satisfactory as it leaves the mouth, to all appearances, unchanged. This operation makes it so that the remaining root or roots do not have to stand more than their share of mastication, and so works for greater stability and a more rapid recovery.

An excellent mouth wash for use at home consists of a saturated aqueous solution of sodium silicifluoride, which amounts to a .61 of 1 per cent. solution. This, when held for a minute in the mouth, morning and evening, and freely swashed through the teeth is a great assistance in relieving inflammation of the gums. It has no perceptible antiseptic coefficient, but, like the biflouride, it seems to restrain the deposition of tartar on the teeth, except in about 1 per cent. of cases, when it seems to slightly increase it. But even in these cases the gums heal promptly, and the superficial deposit is easily removed from the teeth with brush and pumice. The disagreeable flavor of the solution can be disguised by adding an equal amount of sodium chloride or a judicious quantity of aromatics.

An efficient tooth powder that will liberate enough free oxygen to make 40 to 50 drops of a 3 per cent. peroxide solution for every ten grains used on the brush in the mouth is as follows:

| | | |
|---|---|---|
| Peroxide of magnesium (No. 200 mesh sieve) | 60 | parts |
| Perborate of sodium | 30 | " |
| Castile soap | 10 | " |
| Flavoring | | |

This can be used morning and evening with the brush, and should be swashed around the interstices for a full minute before being ejected from the mouth. But, above all, the teeth and gums should be thoroughly cleansed and brushed morning and evening. Without this, antiseptic washes will be of no avail. Floss silk and the toothbrush are the instruments to this end. Since the toothbrush cannot cleanse between the teeth, these surfaces should be swept free from bacterial deposits with floss silk morning and evening, and then the teeth and gums should be thoroughly brushed with strokes not less than an inch and a half long, and rotary wherever possible. The brush should be small, not over an inch and a half long, the bristles not over a quarter of an inch, and narrow, so that when the mouth is partly opened the brush can be placed between the ramus of the jaw and the third molars. Most brushing does not extend beyond the spring of the bristles which, instead of giving bristle friction, merely pivots the bristles without cleansing. The upper and lower third molars more frequently decay, and are subject to pyorrhea alveolaris simply because they are not cleansed. Structurally, they are not weaker than any other teeth.

At each visit of a patient it is most essential that an examination should be made of the necks of all the teeth for bacterial plaques that may have been accumulating undisturbed since the last visit, and these should be pointed out to the patient, and he should be shown what movements of the brush are necessary for their removal, so that in the future he may know how to properly cleanse the teeth and gums. For, a final test of a method of brushing the teeth and gums is, does it clean away the bacterial plaques? Ninety-nine out of a hundred of cleanly people in this world never brush the thick bacterial plaques from their third molars, or, as a matter of fact, from half the other teeth. They simply have never been taught and do not know how. Gums, as well as teeth, should be thoroughly brushed twice a day to clear away all bacterial plaques, and observation of my patients proves that healthy gums are no more injured by vigorous brushing than is the skin adjacent to the finger nails.

Inflamed soft gums will unquestionably be made sore of the first week or two, but persistence on the part of the patient and assistance on the part of the dentist in touching up the sore spots with nitrate of silver will soon strengthen the gums to almost any friction the toothbrush can give them.

The question of vigorous cleansing and massaging the gums with the brush is not only a question of removing external films of infection, it is for the purpose of producing an autoinoculation that will create antibodies in the blood for the purpose of combating the disease, and a vigorous massage of the parts causing a local hyperemia, thus enabling the antibodies in the blood to come into more intimate contact with the infecting bacteria.

This is a most important phase in the cure of pyorrhea, and one that has not been sufficiently emphasized. For the formation of antibodies for the cure of pyorrhea is a means of eradicating pyorrhea from the system. The autoinoculation caused by vigorous gum brushing combined with the judicious use of carefully prepared vaccines have given results of a systemic improvement that are little less than marvelous.

Some two years ago I began the vaccine treatment as an adjunct to my local pyorrhea treatment. I was decidedly skeptical because pyorrhea does not seem to be caused by a specific bacterium, but may be caused by any one of several species, or various combinations of these. Nevertheless, from the very start such increased improvement locally and systemically was obtained that the great value of the treatment could not be doubted. The results in almost every case showed a consistent healing of the pockets and a disappearance of infection from the gums. In about 50 per cent. of the cases there was a distinct improvement in the general physical condition that, as before stated, from being chronic was sometimes made more apparent by its absence than its presence. In the forty cases that are used as the basis of this report, the streptococcus, staphylococcus, bacillus influenza, pneumococcus, micrococcus catarrhalis, diphtheroids, Friedlander bacillus, and an occasional unidentified bacterium were used in the vaccines. The streptococcus viridans appeared in about 90 per cent. of the cases during the winter, but later in the spring and fall it was not found at all. That the vaccines for pyorrhea alveolaris for forty cases contained such a consistent combination of similar germs for so many cases with so comparatively few unidentified strains, may be partly due to the method used in collecting the specimens so as to exclude the incidental or extraneous flora of the mouth.

When the infected area appeared at the tip of a root where the pulp had died, and the root canal had been filled, the culture was always taken through the root canal which was drilled out with a fine sterilized piano wire drill until the end was nearly reached. The canal was then sterilized with carbolic acid, wiped dry with cotton and then blown out with hot air. When this had been accomplished and the tooth had been carefully guarded with a napkin to prevent infection from the mouth, another sterilized drill was passed down to the end, and then plunged through the tip into the infected area. This was then streaked over the blood agar in the ordinary manner.

At times, however, the infected area appeared near the tip of a root or roots where the pulps were alive. In this case the root canal method of obtaining the specimen was not feasible, so the following method was substituted.

The mucous membrane over the indurated infected spot was cocanized, and a thin cautery plunged down to the bone. A sterilized bone drill was then passed through the outer plate of the alveolar process, and the patient dismissed for two days. On his return the opening in the gum on being protected with a napkin, was cauterized with pure carbolic acid and then wiped dry. Then the sterilized point of a small sterilized platinum pointed glass syringe or platinum spear was inserted into the bony opening made previously by the drill, and a small drop of bloody fluid extracted, which with due care was transferred to the blood agar medium. This material was supposed to contain the bacteria that had gathered for the purpose of preventing reorganization of the tissues.

To take a specimen from a pyorrhea pocket, the following method is used: The neck of the tooth and edge of the pocket are first lightly cauterized to destroy all outside bacteria. The tooth is protected from mouth infection by a napkin. Then a small cup-shaped spear of thin platinum having been heated to a cherry red is plunged to the bottom of the pocket so as to get not only the pus in the bottom of the pocket, but also a slight amount of blood from the walls of the cavity. The spear is then withdrawn without touching any other portion of the mouth and streaked over the blood agar. It is thought that the hot platinum will kill any extraneous flora, while the cooled metal will carry to the blood agar only the germs directly responsible for the disease in question. It must not be forgotten that pus is sometimes sterile, while the true cause of infection may lurk within the walls of the abscess from which the material is being obtained.

The vaccine was then made up so as to contain all the germs found, barring the spore formers and the anaerobes that were not grown. While the bacteria of these classes may sometimes play a role in the infection, the results of vaccine treatment indicate that in these cases their role is a minor one. The staphylococci were made to give 300 million to the c.c. Streptococci, diphtheroids, pneumococci, micrococcus catarrhalis, bacillus influenza, and unclassified bacteria were put in so that each would show 50 million to the c.c. In ordinary cases of chronic pyorrhea, the initial dose is 75 million staphylococci and 12 million of each of the other bacteria, and the dose is steadily raised according to the reaction at the site of inoculation and the general systemic response. Where the patient showed exceptional frailness or the inflammation was exceptionally acute, the initial dose was reduced to 37 million staphylococci and 6 million of each of the others. These doses were generally given a week apart in the arm, or, in thin patients, in the back.

Where there is a tendency to the rapid formation of creamy tartar deposits on the teeth prior to vaccine treatment, it will be noted that as the antibodies are formed and the gums show signs of healing, that the tartar will be deposited much less rapidly, and what tartar is deposited is of a more solid removable nature that does not tend to burrow under the gum margin. This change in the deposition of tartar, I have finally come to regard as a distinct symptom of the successful progress of the vaccine inoculations.

When a pyorrhea pocket shows sudden signs of inflammation during treatment, it is always wise to open it surgically with a drill along the root to be sure that there is no back pressure of pus, and that the antibodies have full opportunity to enter the seat of infection for the purpose of aiding in the cure. For, above all things, it should be remembered that the vaccine treatment can only be successful when accompanied by judicious local treatment of a surgical and therapeutic character. And this applies to all foci of infection whether in the gums or in the impaction of a bowel, the genito-urinary system, or anywhere else in the system. Therefore, it is imperative that there should be sympathetic co-operation with the family physician, whose intimate knowledge of the patient, and whose careful diagnosis of foci of infection other than those found in the mouth, will greatly increase the percentage of successes, and add to the permanency of the cures.

A young man with pronounced pyorrhea and several fistulas in the gums came to me so despondent that he thought he was going to die. Local treatment caused improvement, but two of the fistulas would not heal. An autogenous vaccine was made March, 1912, from material obtained from the worst fistula. It contained only a single strain of streptococcus. After four injections, a week apart, he gained in weight, and as he started to slowly lose weight, but as he felt better and the gums improved, the treatment was continued up to June, when there was a great improvement in the gums, and both fistulas had healed. In the fall he returned, and, as he and his family physician said, the vaccine had done him so much good, I made another vaccine from a small pyorrhea pocket, and obtained four strains of streptococci and a staphylococcus aureus. This treatment caused a complete healing of the mouth, so that it was apparently absolutely healthy, his skin cleared, he gained weight, and felt, as he said, well. The mixed vaccine seemed most effective.

He begged me to give the vaccine treatment to his wife, who also was suffering from pyorrhea. She had almost the same experience, except that she did not gain weight (her weight was normal), but her mouth showed great improvement, and she slept better and felt a great lessening of nerve tension.

Mrs. A——, of about fifty years of age, came to me with all the teeth loose, complaining of great fatigue, trouble with the eyes, constipation, and a steady loss of weight. In six months the vaccine treatment had markedly tightened the teeth, and her chronic fatigue and constipation were gone, the wrinkles vanished from her skin, owing to an increase of weight from 115 to 127, and all of her friends asked the cause of her improved condition, which she attributed to the vaccine. Her eyes, she claims, were so improved that she could readily read what before was impossible or accomplished with difficulty.

A married woman of forty, who had been under my local treatment for some two years, took a vaccine about a year ago composed of streptococcus. She had a complexion covered with red patches, gout in the right eye, haemoglobin 67, and great depression with constant fatigue. In six months her haemoglobin went up to 93, the gout disappeared from the eye, her complexion cleared, and the gums made more progress than had previously been made in two years under local treatment. Her greatest satisfaction lay in the fact that she slept well, which, prior to the vaccine treatment, had not been the case.

All my treatments have not been as dramatic or startling as the last two mentioned. However, in three cases as the gums and abscesses healed, marked redness of the nose disappeared. This was associated with great clearing of the complexion and a gain in weight. In other cases there was a consistent healing of the gums that seemed much more rapid than that usually obtained from local treatment alone, but with many of them the time is too short to be sure of a permanence in cure.

In one case that refused to yield either to local or vaccine treatment, a tumor of the breast was discovered. Such a depressing focus was obviously a sufficient reason for the poor results obtained, and as the patient refuses to have an operation the prognosis is not good.

In fairness to local treatment alone, I wish to report a case that is typical of its class. A young doctor came to me suffering from a case of badly infected enlarged tonsil on the right side, which he said he believed came from a pyorrhea infection. A superficial examination of the mouth showed a beautiful healthy set of gums and teeth, but a further investigation with an explorer demonstrated the presence of a deep pyorrhea pocket between the second and third upper right molars. There were also one or two minor pockets on the other side of the mouth. I took the material for an autogenous vaccine, made an application of the bifluoride solution and sent him away. In a week he returned, and to my surprise the tonsil had returned to its normal size and condition. And I am free to admit that it I had used a vaccine I should have given the vaccine the credit for the tonsil's recovery. Later, I used the vaccine, and am treating him now with excellent results. But I mention this case as a caution to those who would underestimate the systemic results that may be obtained by judicious local pyorrhea treatment.

Once more, let me say that vaccines judiciously combined with local treatment seems to be so valuable in the cases of pyorrhea that they should, in my opinion, always be given a fair trial, and that always in sympathetic cooperation with the family physician, who should do everything in his power to remove all depots of general systemic infection, be they in the bowels, the genito-urinary tract, or anywhere else in the body.—Abstract from *Journal of Allied Societies, June 1914.*

